# Outcomes of four-point suture fixated and two-point sutureless posterior chamber IOLs combined with pars plana vitrectomy

**DOI:** 10.1186/s12886-022-02290-5

**Published:** 2022-02-05

**Authors:** Mariya Zyablitskaya, Estee Hong, Royce W. S. Chen, Stanley Chang, Leejee H. Suh

**Affiliations:** Department of Ophthalmology, Edward S. Harkness Eye Institute, College of Physicians and Surgeons, Columbia University, 635 West 165th Street, New York, NY 10032 USA

**Keywords:** Akreos AO60 lens, CT-Lucia 602 lens, Flanged IOL fixation, Gore-tex suture fixation, Pars plana vitrectomy, Posterior chamber IOL, Secondary IOL implantation, Sutureless IOL fixation

## Abstract

**Background:**

While each scleral fixation method has its own advantages, there is a lack of strong evidence to suggest a superior technique. Advances in cataract surgery expand patient eligibility for successful cataract extraction, benefitting a growing population of pseudophakic patients. However, implantation of secondary intraocular lens (IOL) with compromised anterior or posterior capsule is a more challenging task. Each method of scleral fixation has its own advantages and none of them has strong evidence to be superior. This paper describes postsurgical outcomes of two scleral intraocular(IOL) fixation techniques combined with pars plana vitrectomy(PPV) from a single tertiary referral eye center.

**Methods:**

Patients underwent PPV and IOL implantation with either four-point sutured scleral fixation (Akreos AO60(AK); *n* = 24) or two-point sutureless flanged intrascleral fixation (CT Lucia(CTL); *n* = 7). Reports include IOL and sclerotomy placement, fixation techniques, and IOL model.

**Results:**

Thirty-one eyes of thirty patients were analyzed. Average change in vision from baseline measurement was LogMAR − 0.68 ± 0.66 and − 0.90 ± 0.63 for AK and CTL groups, respectively. Average postoperative refractive error was − 0.3 ± 1.03 D (AK) and 0.4 ± 0.60 D (CTL). No opacification cases of Akreos lens were found in this study with the longest follow up of 53 months.

**Conclusions:**

Both methods of implantation (sutured and sutureless) could provide good visual and refractive outcomes. Minimal complication rates were reported despite including patients with multiple comorbidities, making both techniques an attractive choice for secondary IOL implantation.

## Background

The occurrence of a weak posterior and anterior capsule that may require secondary intraocular lens (IOL) fixation is significant considering possible cataract surgery complications (posterior capsule rent, vitreous prolapse), genetic [1, 2], and ocular trauma to a pseudophakic eye [3].

Since the introduction of scleral fixation, the method has undergone several modifications to the following procedural steps: introducing suturing needles—ab externo or ab interno [4], securing the haptic with the fixating suture [5–7], number of fixation points of the scleral fixated posterior capsule IOL (SFPCIOL) [8, 9] and how to avoid suture/knot erosion [10]. Despite several options for implantation such as scleral, iris or sulcus fixated IOL, there is no strong evidence to favor a particular method [11–13]. Iris fixation techniques have been previously utilized in cases without capsular support. Although it is a viable option, there are concerns with iris pigment loss from intraocular lens chafing with this technique. For this reason, many support scleral fixation in aphakic eyes [14]. In patients with multiple ocular comorbidities, available IOL fixation options become restricted. For many patients, vitrectomy is often required in conjunction with implantation of a SFPCIOL. These patients have more unpredictable visual outcomes than uncomplicated patients, and more likely to experience a myopic shift in refraction [11].

The spectrum of patients requiring secondary IOL implantation is diverse in age and comorbidities. Patients may also require combined surgery (glaucoma or corneal), which prolongs surgical time. Therefore, to advance surgical planning and surgical outcomes, it is essential, although challenging, for studies to report surgical techniques and outcomes in such complicated and older patients. This study aims to report the complications rate, as well as visual acuity (VA) and refractive outcomes of the secondary IOL implantation surgery combined with a plana vitrectomy (PPV) from a tertiary referral eye clinic.

## Patients/materials and methods

The study was conducted in accordance with the tenets of the Declaration of Helsinki and has been approved by the Institutional Review Board (IRB) of Columbia University. Due to the retrospective nature of this study, informed consent was waived by the IRB. Electronic medical records of all patients who underwent four-point polytetrafluoroethylene monofilament (PTFE), or Gore-Tex, suture fixation of a foldable Akreos A60 IOL (AK; Bauch and Lomb, Bridgewater, New Jersey, USA) 3 mm behind the limbus and two-point sutureless fixation of a CT-Lucia IOL (CTL; Carl Zeiss Meditec, Jena, Germany) 2.5 mm behind the limbus with concurrent PPV at the Edward S. Harkness Eye Institute, New York, NY from December 2016 through November 2019 were reviewed. Patients were identified from surgical operative reports using the lens catalog number for CTL (formerly EC3-PAL) and AK.

Patients were included in the study if the surgery was performed by one of the vitreoretinal surgeons (R.W.S.C. and S.C.) and corneal surgeon (L.H.S.). Indications for surgery were aphakia with deficient capsular support, dislocated and subluxated IOL or crystalline lens. The following patient data were collected: demographic details, indication for surgery, pre- and post-operative best corrected visual acuity (BCVA), intraocular pressure (IOP) with Tonopen (Reichert technology, NY, USA) and Goldman applanation tonometry preoperative and final postoperative spherical equivalent (SE), ocular comorbidities, intraoperative and postoperative complications. Scleral fixation of IOL in combination with glaucoma drainage valve implant or corneal Descemet’s stripping automated endothelial keratoplasty (DSAEK) were included. Patients with less than 1 month follow up were excluded from the study. In total, data from 30 patients for whom post-operative data were available were selected for the analysis. Pre-operative biometry was carried out using the Zeiss IOL master 700 (Carl Zeiss Meditec, Jena, Germany) or Lenstar LS900 (Carl Zeiss Meditec AG, Jena, Germany) to calculate IOL power for “in-the-bag” position [15]. The target refraction was determined by discussion between the patient and treating surgeon with the usual target of 0. Power calculations were performed using the Barrett formula.

All patients were examined on post-op day 1, week 1 and 1 month with subsequent individualized examination on a case-by-case basis. Snellen VA was converted to logarithm of the minimum angle of resolution (LogMAR). LogMAR equivalents for counting fingers, hand motion and light perception were 1.98, 2.28, and 3, respectively. Refraction was performed and presented for all patients who followed-up and had sufficient VA. Post-operative refractive error (RE) was calculated as final SE minus target refraction. Ocular hypotension was defined as a new onset of IOP of 5 mmHg or less. Ocular hypertension was defined as a new onset of IOP of 25 mmHg or more at any postoperative visit. Corneal edema was documented if found at any postoperative visit, persisting for at least 1 month, and was not observed preoperatively. Cystoid macular edema (CME) was defined as a new onset of postoperative edema that was observed on ophthalmoscopy or optical coherence tomography (OCT). Slit-lamp exam with full pupillary dilation was performed at each visit to estimate the lens position.

### Surgical technique

The technical details for AK and CTL are described in Table 1. Surgical technique was decided by the surgeon after taking the previous surgery into consideration (i.e. Ahmed implant). AK implantation technique using off-label Gore-Tex suture material has been described by Khan [16]. In brief, limited conjunctival peritomies are created at the 3 o’clock and 9 o’clock positions. A toric lens marker is used to mark the cornea at 2 points that are 180° apart with horizontal centration. Then, 2 temporal and 2 nasal sclerotomies are created in a limbus-parallel orientation, 3.0 mm posterior to the limbus, 4 mm apart and straddling the line created by the toric lens marker. In preparation for the AK lens implantation, 8–0 Gore-Tex sutures are passed through paired eyelets on each side of the lens. The lens is folded and inserted through a clear corneal incision and then sutured posterior to the iris plane. The sutures are externalized from each corresponding sclerotomy using intraocular forceps. The sutures are tied in a 3–1-1 fashion to center the IOL, and the knots are rotated into the superior sclerotomies. By comparison, implantation of the CTL utilizes two transconjunctival sclerotomies made with a 27G trocar that are 180° apart, with vertical centration at 12 o’clock and 6 o’clock, 2.5 mm behind the limbus. Once the lens is installed and centered, the haptics are lightly cauterized and pushed into the scleral grooves, as described by S. Yamane 2017 [6]. For all cases, standard 3-port PPV is performed. Both lenses were implanted in the same surgical center by the same surgical teams.

**Table 1:** Surgical characteristics of two-point sutureles (CTL) and four-point sutured (AK) IOL lenses

Surgical characteristics	AK	CTL
Sclerotomy size (G)	25	27
Scleral fixation	3:1:1 GoreTex 8.0	PVDF haptics Flange
Unilateral fixation points distance	4	N/A
Distance from limbus	3	2.5
Long eyes (> 26)	5	2
Short eyes (< 22)	2	1

*PVDF* Polyvinylidene fluoride, *SD* standard deviation

### Statistical analysis

Group differences in baseline characteristics were assessed using the student’s t-test for normally-distributed continuous variables. Normality was tested using Shapiro-Wilk normality test. In cases where non-normality was suspected, statistics were re-calculated using non-parametric test equivalents. In all cases, the non-parametric test results followed the same trend as those reported in the manuscript. Stata software (StataCorp LLC, College Station, Texas) was used to perform the analysis. Significance of all statistical tests were based on an alpha value of 0.05 (*P* ≤ .05).

## Results

A total of 31 eyes of 30 patients (15 females, 15 males) were evaluated. The demographic characteristics are summarized in Table 2. The mean age was 68 ± 19.6 years, ranging from 21 to 95 years. Ocular history included retinal detachment (RD) repair (44% of eyes) and glaucoma (44%; including 5 primary open angle glaucoma, pseudoexfoliative, 1 exfoliative, 1 traumatic, and 1 eye with Uveitis-Glaucoma-Hyphema syndrome). History of corneal pathology was noted in 31% of the eyes: 2 eyes with corneal edema, 4 with bullous keratopathy, and 2 had Fuchs’ endothelial dystrophy. Other ocular history included: CME (19%), age-related macular degeneration (AMD; 19%), globe rupture (6%), and Marfan syndrome (6%). Mean follow up time was 23 ± 15.2 mo. Minimal follow-up was 1 month (for 2 patients), maximum follow up was 53 months. Refraction data was collected at 3 months, not including the previously mentioned 2 patients with 1-month follow-up. All patients underwent PPV. Several patients in the AK group had concurrent surgeries performed: Ahmed valve implant in 4 eyes, and DSAEK in 4 eyes. The latest concurrent surgery included 2 patients with sulfur hexafluoride as a tamponading agent.

**Table 2:** Baseline characteristics of patients selected for the study, including demographic data, ocular comorbidities and past surgical history. Table also includes indication for secondary IOL implantation and concurrent procedure to vitrectomy and IOL implantation

Patient characteristics	Data	
	N	%
Total patients	30	
Total eyes	31	
**Gender**		
Female	15	50
Male	15	50
**Age**		
Mean ± SD	68	±19.62
Range	21–95	
Ocular history		
Glaucoma	14	45
Retinal detachment	14	45
Corneal pathology	8	26
AMD	6	19
Globe rupture	2	6
Cystoid macular edema	6	19
Marfan syndrome	2	6
Previous posterior segment surgery	9	29
Previous anterior segment surgery	6	19
**Indication**		
Dislocated IOL	19	61
Aphakia without capsular support	8	26
Subluxated crystalline lens	4	13
**Concurrent procedure**		
PPV	31	100
Removal of silicone oil	3	10
Membrane peel	3	10
Ahmed implant	4	13
DSAEK	4	13
Akreos	24	77
CT-Lucia	7	23

*SD* standard deviation, *IOL* Intraocular Lens, *DSAEK* Descemet Stripping Automated Endothelial Keratoplasty, *PPV* Pars Plana Vitrectomy, *AMD* Age-related macular degeneration, *IOL* Intraocular Lens

AK was implanted in 24 eyes, and CTL was implanted in 7 eyes. Indications for AK implantation were subluxated artificial lens (14 eyes), aphakia (6 eyes), and subluxated crystalline lens (4 eyes). Indications for CTL implantation were dislocated artificial lens (5 eyes) and aphakia (2 eyes).

### Visual and refractive outcomes

Visual and refractive outcomes are described in Table 3. The mean preoperative BCVA for AK and CTL was 1.31 ± 0.68 D and 1.18 ± 0.84 D, respectively. The final measured BCVA was 0.57 ± 0.62 D (24 eyes) and 0.28 ± 0.26 D (7 eyes) for AK and CTL groups respectively. The average postoperative RE was − 0.3 ± 1.03 D for AK (21 eyes) and 0.4 ± 0.60 D for CTL (7 eyes).

**Table 3:** Preoperative and postoperative visual and refractive outcomes. Baseline and post-operative visual acuity, post-operative refractive error for CTL and AK groups

Visual and refractive outcomes	AK			CTL		
	average	sd	N	average	sd	N
Preoperative BCVA	1.26	±0.66	24	1.18	±0.84	7
Postoperative BCVA	0.57	±0.62	24	0.28	±0.26	7
∆ BCVA*	−0.68	±0.66	24	−0.90	±0.63	7
Average Post operative Refractive Error	−0.30	±1.03	21	0.40	±0.60	7

*BCVA* Best Corrected Visual Acuity

*statistically significant for both groups (*p* < 0.05)

### Complications

Early postoperative complications are notable for 1 eye with CME in the AK and CTL groups each, 2 eyes with haptic erosion in CTL group, and 1 eye with Gore-Tex suture erosion after implantation of the AK. This patient underwent a re-operation with suture repositioning subsequently with no further complications. Ocular hypotension was observed in 2 eyes in the AK group and 2 eyes in the CTL group on post op day 1. A patient from CTL group subsequently developed ocular hypertension on post op week 1. There was one more incidence of ocular hypertension in AK group on post op day one in an eye with a history of glaucoma. CME and hypertension were treated with medications. One of the patients (AK group) had a history of glaucoma. No cases of RD, hemorrhage, lens tilt/dislocation, or lens opacification were reported.

## Discussion

The current study evaluated the visual and refractive outcomes after SFPCIOL implantation with PPV. Despite the high frequency of ocular comorbidities, as well as concurrent surgical procedures in our cohort, the results demonstrated minimal side effects, an improvement of vision, and achievement of refractive goals in the post-operative period.

AK IOL provides a stable four-point fixation with resilient suture material (Gore-Tex), has made this procedure increasingly popular for scleral fixated IOLs. The four-point suture fixation is less prone to tilt, and Gore-tex suture has greater tensile strength and long-term durability than prolene, which reports up to 27.9% suture erosion in adults at a mean follow-up of 6 years [9, 17, 18]. It is, however, associated with increased surgical time due to greater technical complexity and reported sporadic cases of lens opacification [19–21]. In contrast, sutureless IOL implantation inherently avoids suture-associated complications, such as suture degradation and IOL dislocation, and shortens surgical time. Results of a two-point sutureless flanged intrascleral fixation technique reported by Yamane demonstrated good visual and refractive outcomes with a stable IOL location after a maximum follow up of 36 months.

Flanged IOL fixation with a double-needle technique presented by Yamane with four different lenses positioned 2 mm behind the limbus (*n* = 97) notably reported no IOL dislocation or haptic related scleral fixation issues after over 3 years of follow up [6]. The most common postoperative complication reported by the referenced study [6] was iris and vitreous capture in 8 and 5% of the patients, respectively, which can possibly be avoided by placing the lens farther from the limbus. In this study, the lens was positioned 2.5 mm behind the limbus, and no postoperative hemorrhage cases were reported. Our study demonstrated favorable outcomes for combined PPV with two different secondary IOL implantation techniques: four-point Gore-tex suture fixated IOL positioned 3.0 mm behind the limbus, and 2-point sutureless IOL positioned 2.5 mm behind the limbus.

Significant improvements in visual outcomes (*p* < 0.05), despite multiple comorbidities, were shown for both lenses. Patient groups for the two lenses had similar baseline VA. However, the mean final BCVA was better for the CTL group, which is most likely due to lesser prevalence of comorbidities. For secondary IOL AK implantation [11, 22, 23] and flanged IOL fixation [24], similar visual and refractive outcomes were reported taking into consideration common ocular history of patients which included RD repair, glaucoma, trauma with and without globe rupture, complicated cataract surgery [8, 25, 26]. The likelihood of certain postoperative complications may be linked to specific surgical procedures, and the degree of improvement in visual outcomes may be linked to the patient’s ocular history [25]. The majority of the patients had one or more ocular comorbidities (75%) and underwent concurrent surgical procedure (22%), which is more than reported in other studies [16, 23] and some had longer surgical times for cases involving combined corneal and glaucoma surgeries. Despite that, our patients experienced relatively fewer complications in the postsurgical period.

Preoperative refractive goals were achieved by the majority of patients in our study, however, the change was not statistically significant. Similar refractive outcomes have been reported by others implanting SFPCIOL, with variable refractive outcomes: more myopic outcomes reported by Terveen [22] and Fass [27], and slightly hyperopic outcomes reported by Yang and Abbey despite the SFPCIOL being positioned 1.5 mm and 2 mm behind the limbus respectively [28]. A study by Brunin et al. found that SFPCIOL had the most myopic refractive outcomes when compared to the combination of iris, anterior chamber and sulcus fixated IOLs [11]. Our study reported slightly hyperopic outcomes in CTL group compared to the AK group. This could represent the agreement with previously published results as mentioned above, or variability due to small sample size. Notably, the average difference in refractive values were within ±0.5D between the groups.”

The most common complication in this study was ocular hypotension in 4 eyes (2 patients in AK group, 2 patients in CTL group). Three out of four patients in whom it was detected had a previous history of ocular surgery, underwent a concurrent DSAEK procedure, and had a history of glaucoma. Hypotension did not extend beyond the post-operative week 1. While the source of this finding is not clear, incompetence of the self-sealing sclerotomies may be the cause. During the study period, a change in the surgical technique for the sclerotomies was created from radial sclerotomies to limbus-parallel incisions. This change appeared to reduce the incidence of postoperative hypotony. No lens destabilization was reported in the follow up period for any of the patients.

Various methods of fixation might be considered in patients with aniridia and cases with globe rupture. Patients in presented study, however, were successfully treated with the mentioned techniques.

Alternatively, good cosmetic and refractive outcomes can be achieved in patients with globe rupture aniridia and aphakia using a pigmented diaphragm IOL. However, the major disadvantage of such lenses is requirement for larger sclerotomy size and subsequent risk of corneal decompensation postoperatively [29].

One of the concerns implanting any hydroxyethyl methacrylate (HEMA) IOLs such as AK is opacification. Unlike foldable silicone lens opacification occurring intraoperatively, HEMA lens opacification can occur late in the postoperative period [30]. Although quite rarely observed, opacification can occur at any time as early as 2 months and as late as 24 months post-operatively [19, 31]. Opacification is reported to be caused by crystalline deposits and is associated with any exposure to air such as gas tamponade or combined surgery with DSAEK and Descemet’s membrane endothelial keratoplasty (DMEK) [32]. In this study, there were no cases with AK opacification, despite several patients undergoing concomitant Akreos fixation and DSAEK with gas tamponade with the longest follow up of over 4 years.

Both PFTE sutured and flanged haptic sutureless methods carry a risk of suture erosion. In our study, haptic extrusion in sutureless flanged haptic IOL fixation occurred in two patients. Usage of appropriate haptic material to create a proper flange shape and size is critical. In a technique described by Yamane, the haptics are made of polyvinylidene fluoride (PVDF), although some IOLs used in the study have restricted commercial availability in the United States. CTL’s use same PVDF material, which has a melting temperature around 177 °C. Less heat resistant materials, such as PMMA (melting temperature around 110 °C), are not able to consistently provide appropriate haptics [33], increasing lens susceptibility to dislocation as reported by Stem using 3-piece (Alcon) IOLs with PMMA haptics.

Several limitations of this study can be noted. The retrospective design restricted patient selection to those only with existing visual and refractive data. Inconsistent follow-up and small group sizes decreased the power of analysis and statistical significance. It is worth noting that we can only report our experience with two techniques but not compare them to each other. Too many variables, such as comorbidities and previous surgical history, unavoidably made the outcomes difficult to reliably compare. In certain patients, severe late stages of the comorbidities such as glaucoma prohibited the evaluation of visual outcomes related to the refractive surgery. It is also much more difficult to predict the refractive outcomes due to comorbidities. Despite the differences in the lens implantation technique, however, surgical procedure from the same ophthalmic center allows to account for intangible differences between the two.

Our report demonstrated good visual and refractive outcomes, as well as minimal side effects occurring in the post-operative period, despite patients having multiple ocular comorbidities and concurrent surgeries. Although there are differences in surgical technique and specifications, surgical techniques discussed provide good visual and clinical outcomes and are equally well tolerated by patients with multiple comorbidities. The selection of one technique over another certainly depends on the surgeon preferences and skills, as well as patient factors. Advantage of each technique should be carefully weighed for each patient. For example, shorter surgical time for CTL and more stable fixation with 4 points for AK group may be considered in surgical planning. Careful selection of surgical technique and concurrent surgery planning can lead to excellent visual and refractive outcomes accompanied by a low incidence of postoperative complications. Additional studies should be performed to include the newest fixation techniques and expand on established procedures.

## Conclusions

Our study evaluated long term outcomes of sutureless and sutured SFPCIOL techniques. No significant long term side effects were found in neither implantation technique. No tilt or dislocation were reported in this study using CTL two-point fixation lens. Concerns for Akreos lens opacifications reported previously were not supported in this study, suggesting that the defining factors in selecting IOL models is mainly surgical technique preference.

## Data Availability

The datasets used and/or analysed during the current study are available from the corresponding author on reasonable request.

## Abbreviations

IOL: Intraocular Lens
PPV: Pars plana vitrectomy
PTFE (Gore-Tex): Polytetrafluoroethylene monofilament
AK: Akreos AO60 lens
CTL: CT-Lucia lens
BCVA: Best Corrected Visual Acuity
LogMAR: Logarithm of Minimal angle of Resolution
RE: Refractive Error
CME: Cystoid Macular Edema
SFPCIOL: Scleral Fixated Posterior Chamber IOL
IOP: Intraocular tonometry
AMD: Age related macular degeneration
Sph: Spherical power (D)
PMMA: Polymethyl methacrylate
HEMA: Hydroxyethyl methacrylate
DSAEK: Descemet’s stripping automated endothelial keratoplasty
DMEK: Descemet’s membrane endothelial keratoplasty
PVDF: Polyvinylidene fluoride

## References

[1] Karger RA, Jeng SM, Johnson DH, Hodge DO, Good MS (2003). Estimated incidence of pseudoexfoliation syndrome and pseudoexfoliation glaucoma in Olmsted County, Minnesota. J Glaucoma.

[2] Sadiq MA, Vanderveen D (2013). Genetics of ectopia lentis. Semin Ophthalmol.

[3] Channa R, Zafar SN, Canner JK, Haring RS, Schneider EB, Friedman DS (2016). Epidemiology of eye-related emergency department visits. JAMA Ophthalmol.

[4] Lewis JS (1991). Ab externo sulcus fixation. Ophthalmic Surg.

[5] Prenner JL, Feiner L, Wheatley HM, Connors D (2012). A novel approach for posterior chamber intraocular lens placement or rescue via a sutureless scleral fixation technique. Retina.

[6] Yamane S, Sato S, Maruyama-Inoue M, Kadonosono K (2017). Flanged Intrascleral intraocular Lens fixation with double-needle technique. Ophthalmology.

[7] Khan MA, Gerstenblith AT, Dollin ML, Gupta OP, Spirn MJ (2014). Scleral fixation of posterior chamber intraocular lenses using gore-tex suture with concurrent 23-gauge pars plana vitrectomy. Retina.

[8] Botsford BW, Williams AM, Conner IP, Martel JN, Eller AW (2019). Scleral fixation of intraocular lenses with Gore-Tex suture: refractive outcomes and comparison of Lens power formulas. Ophthalmol Retina.

[9] Vote BJ, Tranos P, Bunce C, Charteris DG, Da Cruz L (2006). Long-term outcome of combined pars plana vitrectomy and scleral fixated sutured posterior chamber intraocular lens implantation. Am J Ophthalmol.

[10] Por YM, Lavin MJ (2005). Techniques of intraocular lens suspension in the absence of capsular/zonular support. Surv Ophthalmol.

[11] Brunin G, Sajjad A, Kim EJ, Montes de Oca I, Weikert MP, Wang L, Koch DD, Al-Mohtaseb Z (2017). Secondary intraocular lens implantation: complication rates, visual acuity, and refractive outcomes. J Cataract Refract Surg.

[12] Schein OD, Kenyon KR, Steinert RF, Verdier DD, Waring GO, Stamler JF, Seabrook S, Vitale S (1993). A randomized trial of intraocular lens fixation techniques with penetrating keratoplasty. Ophthalmology.

[13] Stem MS, Todorich B, Woodward MA, Hsu J, Wolfe JD (2017). Scleral-fixated intraocular lenses: past and present. J Vitreoretin Dis.

[14] Toro MD, Longo A, Avitabile T, Nowomiejska K, Gagliano C, Tripodi S, Choragiewicz T, Kaminska A, Figus M, Posarelli C (2019). Five-year follow-up of secondary iris-claw intraocular lens implantation for the treatment of aphakia: anterior chamber versus retropupillary implantation. PLoS One.

[15] Ortiz A, Galvis V, Tello A, Viana V, Corrales MI, Ochoa M, Rodriguez CJ (2019). Comparison of three optical biometers: IOLMaster 500, Lenstar LS 900 and Aladdin. Int Ophthalmol.

[16] Khan MA, Rahimy E, Gupta OP, Hsu J (2016). Combined 27-gauge pars Plana Vitrectomy and scleral fixation of an Akreos AO60 intraocular Lens using Gore-Tex suture. Retina.

[17] Price MO, Price FW, Werner L, Berlie C, Mamalis N (2005). Late dislocation of scleral-sutured posterior chamber intraocular lenses. J Cataract Refract Surg.

[18] Parekh P, Green WR, Stark WJ, Akpek EK (2007). Subluxation of suture-fixated posterior chamber intraocular lenses a clinicopathologic study. Ophthalmology.

[19] Cao D, Zhang H, Yang C, Zhang L (2016). Akreos adapt AO intraocular lens opacification after vitrectomy in a diabetic patient: a case report and review of the literature. BMC Ophthalmol.

[20] Kalevar A, Dollin M, Gupta RR (2020). Opacification of scleral-sutured Akreos Ao60 intraocular Lens after Vitrectomy with gas Tamponade: case series. Retin Cases Brief Rep.

[21] Gregori NZ, Echegaray JJ, Flynn HW (2019). Opacification of Akreos hydrophilic acrylic Lens after retinal detachment repair with silicone oil Tamponade: a case report. Ophthalmol Ther.

[22] Terveen DC, Fram NR, Ayres B, Berdahl JP (2016). Small-incision 4-point scleral suture fixation of a foldable hydrophilic acrylic intraocular lens in the absence of capsule support. J Cataract Refract Surg.

[23] Su D, Stephens JD, Obeid A, Borkar D, Storey PP, Khan MA, Hsu J, Garg SJ, Gupta O (2019). Refractive outcomes after pars Plana Vitrectomy and scleral fixated intraocular Lens with Gore-Tex suture. Ophthalmol Retina.

[24] Yokogawa H, Kobayashi A, Okuda T, Mori N, Masaki T, Sugiyama K (2018). Combined Keratoplasty, pars Plana Vitrectomy, and flanged Intrascleral intraocular Lens fixation to restore vision in complex eyes with coexisting anterior and posterior segment problems. Cornea.

[25] Botsford B, Williams AM, Conner IP, Eller AW, Martel JN. Scleral Fixation of Posterior Chamber Intraocular Lenses Using Gore-Tex Suture: Clinical Outcomes From a Single Institution. Journal of VitreoRetinal Diseases. 2018;2(5):276-81. 10.1177/2474126418791701.

[26] Nowomiejska K, Haszcz D, Onyszkiewicz M, Choragiewicz T, Czarnek-Chudzik A, Szpringer-Wabicz A, Baltaziak K, Brzozowska A, Toro MD, Rejdak R (2021). Double-needle Yamane technique using flanged Haptics in ocular trauma—a retrospective survey of visual outcomes and safety. J Clin Med.

[27] Fass ON, Herman WK (2010). Four-point suture scleral fixation of a hydrophilic acrylic IOL in aphakic eyes with insufficient capsule support. J Cataract Refract Surg.

[28] Yang JM, Yoon KC, Ji YS (2015). Transscleral fixation of single-piece foldable acrylic lens with eyelets at the optic-haptic junction. Can J Ophthalmol.

[29] Chorągiewicz T, Nowomiejska K, Haszcz D, Nowakowska D, Avitabile T, Reibaldi M, Jünemann AGM, Toro MD, Rejdak R (2019). Transscleral fixation of black diaphragm intraocular Lens in complete Aniridia and Aphakia due to posttraumatic eye rupture: a pilot study. J Clin Med.

[30] Jensen MK, Crandall AS, Mamalis N, Olson RJ (1994). Crystallization on intraocular lens surfaces associated with the use of Healon GV. Arch Ophthalmol.

[31] Werner L, Wilbanks G, Nieuwendaal CP, Dhital A, Waite A, Schmidinger G, Lee WB, Mamalis N (2015). Localized opacification of hydrophilic acrylic intraocular lenses after procedures using intracameral injection of air or gas. J Cataract Refract Surg.

[32] Giers BC, Tandogan T, Auffarth GU, Choi CY, Auerbach FN, Sel S, Mayer C, Khoramnia R (2017). Hydrophilic intraocular lens opacification after posterior lamellar keratoplasty - a material analysis with special reference to optical quality assessment. BMC Ophthalmol.

[33] Kronschlager M, Blouin S, Roschger P, Varsits R, Findl O (2018). Attaining the optimal flange for intrascleral intraocular lens fixation. J Cataract Refract Surg.

